# Evidence-based Prostate Cancer Screening Interventions for Black Men: A Systematic Review

**DOI:** 10.1007/s40615-024-02085-y

**Published:** 2024-07-10

**Authors:** Abigail Lopez, Jared T. Bailey, Dorothy Galloway, Leanne Woods-Burnham, Susanne B. Montgomery, Rick Kittles, Dede K. Teteh-Brooks

**Affiliations:** 1 Department of Health Sciences, Crean College of Health and Behavioral Sciences, Chapman University, One University Drive, Orange, CA USA; 2 Gangarosa Department of Environmental Health, Rollins School of Public Health, Emory University, Atlanta, GA USA; 3 River Oak Center for Children, Sacramento, CA USA; 4 School of Behavioral Health, Loma Linda University, Loma Linda, CA USA; 5 Department of Surgery, Morehouse School of Medicine, Atlanta, GA USA; 6 Department of Community Health and Preventive Medicine, Morehouse School of Medicine, Atlanta, GA USA

**Keywords:** Prostate cancer, Racial health disparities, Cancer screening, Clinical trial participation, Black men

## Abstract

**Abstract:**

Prostate cancer is the second leading cause of death for men in the U.S. and Black men are twice as likely to die from the disease. However, prostate cancer, if diagnosed at an earlier stage, is curable. The purpose of this review is to identify prostate cancer screening clinical trials that evaluate screening decision-making processes of Black men.

**Methods:**

The databases PubMed, Ovid MEDLINE, CINAHL Plus, and PsychInfo were utilized to examine peer-reviewed publications between 2017 and 2023. Data extracted included implementation plans, outcome measures, intervention details, and results of the study. The Critical Appraisal Skills Programme was used to assess the quality of the evidence presented.

**Results:**

Of the 206 full-text articles assessed, three were included in this review. Educational interventions about prostate cancer knowledge with shared and informed decision-making (IDM) features, as well as counseling, treatment options, and healthcare navigation information, may increase prostate cancer screening participation among Black men. Additionally, health partner educational interventions may not improve IDM related to screening participation. The quality of the evidence presented in each article was valid and potentially impactful to the community.

**Discussion:**

Black men face various social determinants of health barriers related to racism, discrimination, cost of health services, time away from work, and lack of trust in the healthcare system when making health-related decisions, including prostate cancer screening participation. A multifactorial intervention approach is required to address these inequities faced by Black men especially as prostate cancer is curable when diagnosed at an earlier stage.

**Supplementary Information:**

The online version contains supplementary material available at 10.1007/s40615-024-02085-y.

Prostate cancer is the second leading cause of death for men in the United States (U.S.) [1]. Incidence rates for prostate cancer have increased since 2014 despite declines noted in previous years (2007–2014) [1]. While the diagnosis of localized prostate cancer initially declined in the years following the discontinuance of blanket annual screening in 2012 at the recommendation of the U.S. Preventive Services Task Force, the diagnosis of regional and metastatic prostate cancer increased [2]. Many American men avoid seeking healthcare-related services due to various social and environmental determinants [3] including masculinity ideals and socialization [4]. In turn, a delay or avoidance in seeking healthcare related services can lead to a later-stage diagnosis, thus reducing the likelihood for treatment and ultimately survival. Screening tools can detect cancer in its early stages and often require less intensive treatment and are known to increase survival [5]. The American Cancer Society encourages men (age 50 for men at average risk, age 45 for men at high risk, and age 40 for men at even higher risk) to discuss their options and learn about the potential advantages of prostate cancer screening [1, 6, 7].

Generally, healthcare delivery clinical trial interventions are utilized by providers and researchers to identify novel ways to prevent a disease or condition [8]. This approach can support the understanding of factors that influence screening and research participation among various racial and ethnic population groups. However, the lack of diversity in clinical trials creates obstacles for addressing health concerns especially among communities of color [9, 10]. For instance, rates for prostate cancer differ among racial/ethnic groups with Black men having the highest incidence rates of prostate cancer in the U.S. [11]. Black men are twice as likely to die from the disease compared to White men [12]. Thus, it is important that Black men are represented in clinical trial interventions to identify effective strategies to prevent incidence of prostate cancer.

Despite the urgent need for preventive screening, Black men’s mistrust in academic and research institutions rooted in structural racism, discrimination, and historical mistreatment by health professionals present significant barriers to adopting screening behavior [13–16]. Other factors such as cost of health services, time away from work, and societal norms about masculinity are notable barriers and concerns that Black men are confronted with when making health-related decisions such as prostate cancer screening participation [17–19].

The rates for prostate cancer continue to rise, approximately 12% of men will receive a diagnosis during their lifetime, and Black men are disproportionately impacted by the disease [1]. This disparity emphasizes the need to educate Black men about prostate cancer screening and encourage informed decision-making behaviors [20]. To address health-related decision-making factors and processes experienced by Black men, a systematic literature review was conducted to identify clinical trial behavioral interventions. The following review has been prepared in compliance with the criteria outlined in the Preferred Reporting Items for Systematic Reviews and Meta-Analyses (PRISMA) checklist [21].

## Methods

### Eligibility Criteria

Inclusion guidelines included peer-reviewed full text articles, published between 2017 and 2023, conducted in the U.S., English language, with Black/African American men above 40 years, reported qualitative and quantitative outcomes, and followed a randomized control trial design. Studies with younger below 39 years, non-African American/Black men were eliminated from this analysis. We also excluded observational and non-interventional trials, studies conducted outside of the U.S., published prior to 2017, non-English studies, dissertations, non-peer review manuscripts, meta-analyses, and other systematic reviews.

### Search Strategy and Selection Process

**Fig. 1:**
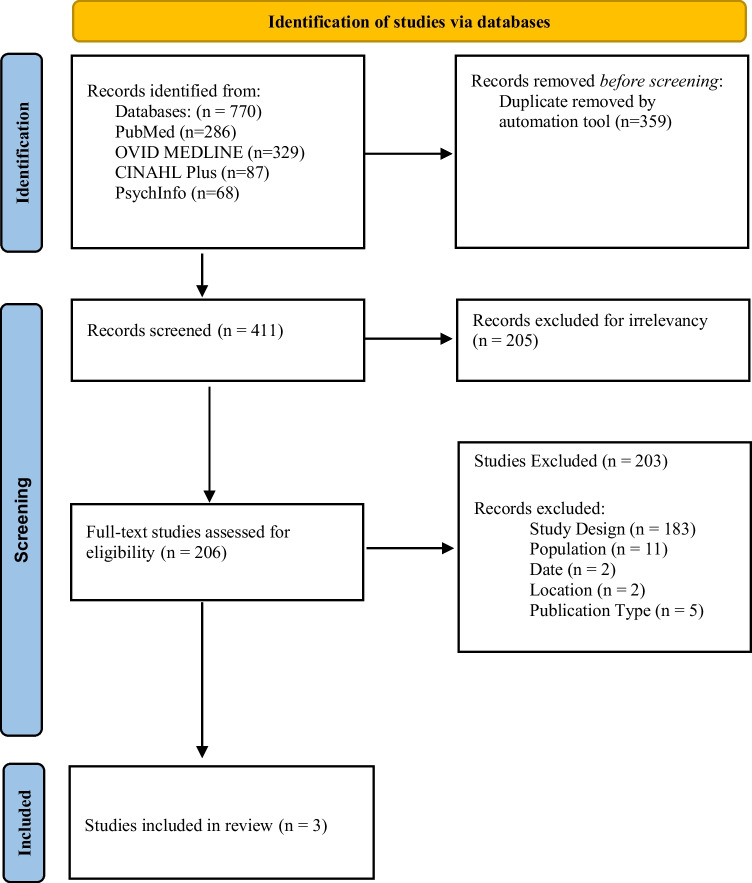
PRISMA flow diagram included the number of publications at identification, screening and final publications for data extraction and analysis

The authors collaborated with a librarian to develop a search strategy criterion using the four online databases: PubMed, Ovid MEDLINE, CINAHL Plus, and PsychInfo. The librarian completed two search strategies for this review. These searches generated 770 articles (see Appendix A and Appendix B for the full search strategies). After removal of duplicates [22], 411 articles were included in the title/abstract evaluation. In the next phase, 205 articles were removed for not matching the inclusion criteria, leaving 206 articles for the full-text analysis. These articles were further evaluated based on inclusion criteria (see Fig. 1), 203 articles were excluded due to study design (*n* = 183), population/ target audience (*n* = 11), publication date (*n* = 2), location of study (*n* = 2), and publication type (*n* = 5). After the title/abstract and full-text screening, three articles were included in this review.

### Data Abstraction

Covidence systematic review software management tool [22] was used to conduct the title/abstract and full-text screening. Co-authors DG, JB, and AL participated in the title/abstract screening and JB and AL participated in the full-text screening. Title/abstract and full-text screening were completed independently. Disputes related to article inclusion were addressed through covidence via the notes section and discussed during team meetings. The team utilized a data extraction template developed by AL to collect data from each article. The data from the extraction template included general information (title of study, date published, link to article, location the study), aim of study, study design, inclusion/exclusion criteria, recruitment and retention strategies, intervention details (intervention group names and descriptions, total number of participants randomly assigned to each group, description of intervention delivery, outcome measures), and study findings.

### Quality Assessment

The Critical Appraisal Skills Programme (CASP) [23] was used by JB and AL to assess the quality of the evidence provided for each article. CASP is an 11-item quality assessment tool that involves prompt questions with “Yes,” “No,” and “Can’t tell” responses to help evaluate research studies. It includes screening questions about the validity of the basic study design, how the study was executed (to determine systematic errors and bias), and the potential impact of the results to the community. JB and AL rated the articles independently. High-quality articles were found to include the following: a clearly focused research question, random assignment to the intervention, and study groups with limited characteristic differences that could alter the outcome of the intervention. The research team provided the same level of care to all intervention groups, reported the effects of the intervention comprehensively, and reviewed if the results can be applied at the community level.

## Results

Of the 206 full-text articles assessed for eligibility, three were included in this review [17–19]. As shown in Table 1, two of the three clinical trial interventions were conducted in a healthcare setting (cancer center or clinical facility/site) and one study was conducted in a community setting (churches). Two studies included both Black and non-Hispanic white male participants. All studies had a randomized control trial design focused on identifying methods to increase IDM for prostate cancer screening participation and all included Black men in the U.S.

**Table 1:** Participant and study characteristics

Author and year	Participant characteristics			Study characteristics				Retention techniques	Outcome measures
	Age	Race/ethnicity	Status	Study design	Sample size	Location	Method of recruitment		
Screening interventions at health care facilities									
Roussi 2018	35–69	AA & C	No cancer	Two-arm RCT	128	PA, USA	Prostate Risk Assessment Program (PRAP)	None	Knowledge, perceived risk, positive and negative screening expectancies, and intrusive ideation
Carlson 2021	18–40+	AA and others^a^	No cancer	RCT	175	OH, USA	Radio, news announcements and flyers disseminated at local community centers, churches, and church groups.	None	Decisional conflict and knowledge improvement
Screening intervention at community setting									
Holt 2017	18+ with mean age 56.18	AA	No cancer	Cluster RCT	262	USA	Churches, Community Health Advisors (CHAs) Incentives: Enrolled men received a $25 store gift card for each workshop attendance and survey completion.	Text messages, quarterly newsletters, and $25 gift card incentives for follow-up surveys.	IDM, knowledge, and screening

*RCT* randomized control trial, *IDM* informed decision making, *Race/Ethnicity* C-Caucasian, *AA* African American, *Others (5 or less people for each group)* Caucasian, Asian/Pacific Islander, other

### Screening Interventions at Healthcare Facilities

Roussi and colleagues [17] explored the psychosocial impact of enhanced counseling versus a general health education intervention at a comprehensive cancer center in Pennsylvania. The intervention aimed to prepare participants for potential consequences of receiving prostate cancer screening test results. The men assigned to the counseling arm engaged in role-play scenarios that included their reactions to the receipt of various screening results. Men assigned to the general health education intervention received both information about men at high risk of prostate cancer and health recommendations such as smoking, alcohol abuse, diet, and exercise. Additionally, men assigned to the general health education arm were also encouraged to discuss their personal attitudes, beliefs, expectations, and emotions throughout the process. Participants were assessed at three time periods throughout the intervention study: baseline, 3 weeks post-intervention, and 6 months post-intervention. The outcome measures included knowledge of prostate cancer, perceived risk, positive and negative expectancies, and intrusive ideation related to risk. Black men who received the counseling intervention reported higher levels of perceived risk of disease and reported more negative expectancies than their White counterparts. Black men in the education group were more likely to have higher intrusive ideations compared to men in the counseling group. At the 6-month follow-up, Black men were more likely than White men to report negative beliefs related to prostate cancer screening including fears of discrimination, time, and cost for screening.

Carlson and colleagues [18] examined an educational intervention with added components of shared versus IDM among Black men at a clinical facility in Ohio. This study was modified from the team’s previous single arm intervention that was divided into three multi-step components (education for IDM, screening and follow-up. The study was modified to a two-arm study: IDM compared to SDM (shared decision-making). IDM was defined as the understanding of the disease, the outcomes, and ultimately, a decision that aligns with one’s own values. SDM was defined as a collaboration between the individual and a healthcare professional to make a healthcare related decision. The intervention (SDM) and control groups (IDM) received a 20-min presentation on prostate cancer screening risks, benefits, consequences, and alternative options. Healthcare providers were involved in development of the pre- and post-screening assessment tool to assess knowledge of prostate cancer but were not included in the decision tool portion of the study.

Both the IDM and SDM intervention groups received the same educational presentation, but the SDM arm received a decisional tool embedded into the presentation to review before deciding whether to get screened. Participants had the option to undergo prostate-specific antigen (PSA) test and a digital rectum examination (DRE), not to be screened, or could reply with being “unsure.” Individuals were also able to opt out of the education component completely. Men over 18 were eligible for the educational component while only men over 40 were eligible for the educational and screening components. The outcome measures included decisional conflict about prostate cancer screening and prostate cancer knowledge. There was an increase in prostate cancer screening knowledge among both groups, and no significant changes in uncertainty or conflict for deciding to undergo screening. The total number of individuals 40 or older who decided to participate in screening was not reported, but 69 (87.34%) individuals from the IDM arm and 89 individuals (86.46%) from the SDM arm indicated that they desired to get screened for prostate cancer. Six individuals in each group decided that they would prefer a doctor to make the decision for them.

### Screening Intervention at Community Setting

Holt and colleagues [19] evaluated a health partner education intervention to increase IDM for prostate cancer screening among Black men in a church setting. Two groups were included in the intervention: men only or men and their female health partner. The intervention was a four-part educational series that included information about prostate cancer screening, IDM for screening, treatment options, and navigating their healthcare team. Each session incorporated interactive discussions with opportunities for questions and answers. The primary outcome measure was IDM, which was assessed using the Stage of Decision-Making Scale [24], which included response ranges from “haven’t begun to think about making a decision,” “started to think about my decision,” and “already made my decision,, as well as the role of decision-making (e.g., who should make the decision versus who made the decision), and preparation of decision-making (e.g., if the intervention helped prepare them for making a decision). Results indicated including health partners in the intervention did not improve IDM among participants. The study had significant improvements in stage of decision making among men who attended majority of the four sessions, which indicates less conflict (higher intentions) with coming to a decision about screening.

### Quality Assessment

The Critical Appraisal Skills Programme (CASP) [23] 11-item quality assessment tool was used to assess the validity of the basic study design, study implementation, and the potential impact of the results to the community (see Table 2). All three articles included in this review addressed a clearly focused research question, had randomized intervention assignment, and included results that can be used to inform future community interventions. However, it was unclear if the research investigators were 'blind' to the intervention they were giving to participants.

**Table 2:** The Critical Appraisal Skills Programme (CASP) quality assessment

Question	Author and year		
	Roussi (2018)	Carlson (2021)	Holt (2017)
1. Did the study address a clearly focused research question?	Yes	Yes	Yes
2. Was the assignment of participants to interventions randomized?	Yes	Yes	Yes
3. Were all participants who entered the study accounted for at its conclusion?	No	No	No
4. Were the participants “blind” to intervention they were given?	Yes	Yes	Yes
5. Were the investigators “blind” to intervention they were giving to participants?	Can’t tell	Can’t tell	Can’t tell
6. Were the study groups similar at the start of the randomized controlled trial?	Yes	Yes	Yes
7. Apart from the experimental intervention, did each study group receive the same level of care (that is, were they treated equally)?	Yes	Yes	Yes
8. Were the effects of intervention reported comprehensively?	Yes	Yes	Yes
9. Was the precision of the estimate of the intervention or treatment effect reported?	Yes	Yes	Yes
10. Do the benefits of the experimental intervention outweigh the harms and costs?	Yes	Yes	Yes
11. Can the results be applied to your local population/ in your context?	Yes	Yes	Yes

## Discussion

In the U.S., Black men are twice as likely to die from prostate cancer than non-Hispanic White men [12] and continue to be underrepresented in clinical trials [25]. This results in limited understanding about Black men’s overall perceptions and barriers to prostate cancer screening. This is framed in the context of racism, discrimination, cost of health services, time away from work, lack of trust in the healthcare system, and societal norms about masculinity, all of which are additional barriers that Black men may be confronted with when making health-related decisions [17–19]. This systematic literature review assessed three randomized clinical trials focused on the decision-making process for prostate cancer screening among Black men.

Black men’s personal experiences can provide insight into the barriers that they may face when engaging with medical providers and the healthcare system. The intervention conducted by Roussi and colleagues [17] located at a healthcare facility in Pennsylvania found that an enhanced counseling intervention impacted Black and non-Hispanic White men differently. Black men reported higher levels of perceived risk and more negative psychological responses to making the decision to screen, including fear of discrimination, time, and cost. This emphasizes the importance of culturally tailored interventions, to address barriers that Black men face when choosing to proceed with prostate cancer screening. Educational materials with culturally sensitive information (including transparency about the history of the negative healthcare experiences with the Black population) [26] and patient centered communication such as jargon free information, shared power in decision-making, and active listening may improve relationships between providers and Black men [27, 28]. Black men are also concerned about cost of screening and time away from work [17]. These concerns highlight some of the health-related decision-making barriers to prostate cancer screening among Black men in the U.S.

In the educational intervention conducted by Carlson and colleagues [18], the team tested incorporating shared versus IDM components. The intervention was effective in increasing knowledge about prostate cancer screening; however, the decision tool embedded into the SDM presentation did not address feelings of uncertainty about the decision to undergo screening. While the results from the intervention [18] indicate a presentation covering prostate cancer screening risks, benefits, consequences, and alternative options could increase knowledge about prostate cancer, it did not address the psychosocial aspects of the decision-making process to screen for the disease. Healthcare providers were included in the development of the evaluation tools but did not interact with participants before their decision. Trust and communication between the healthcare professional and patient is essential for fostering trust [29]. Perceived medical mistrust between the patient and healthcare professional is a significant barrier to quality care and has shown to negatively influence patient behavior such as reduced self-efficacy to adhere to treatment plan, which often leads to undesired health outcomes [30]. Future studies examining the relationship and communication between the patient and healthcare providers are warranted to identify barriers to healthcare service utilization among Black men such as discrimination, racism, as well as negative perceptions and ideations about the health system and providers.

Furthermore, while partners may play a role in Black men’s general health-related behaviors, it may not lead to increased participation in prostate cancer screening [19]. Holt and colleagues faced challenges with incorporating health partners in their clinical trial intervention. However, participants in the health partner intervention group were not asked to provide details on issues or concerns they experienced when asked to bring a health partner to the session. Additionally, the inclusion of health partners did not improve IDM among participants. A probable explanation may be the differences in social connection and expression of social need between men and women [31]. When compared to women, men tend to have limited social networks and are less inclined to seek out social support. Some men may also have difficulty seeking assistance due to their lack of knowledge of effective ways to ask for support. Black men are even more likely than non-Hispanic white men to internalize the masculinity ideology (e.g., restricting emotion) [32], therefore making them less likely to include a partner in the vulnerability of considering prostate cancer screening, which, if illness is indeed identified, may impact their masculinity. However, we know that Black men are open to seeking informal social support by going to barbershops or churches [32]. The use of community assets such as barbershops has been extensively researched and has shown to address health disparities in Black men [33]. In turn, creating an inclusive culturally tailored environment for Black men to have conversations and express vulnerability about possible risk to their sexuality if they are found to have prostate cancer may encourage more men to discuss their fears, concerns, ask questions, and receive social support for making a life confirming decision if needed. Encouraging men to openly discuss their health may challenge societal norms about masculinity and foster an environment that may empower Black men to actively engage in their overall well-being including engaging in prostate cancer screening behaviors.

### Practice, Policy, and Future Research

There are complexities associated with prostate cancer screening adherence for Black men [3]. The interplay of intrapersonal (individual), interpersonal (relationships), community, and societal (racism, discrimination, healthcare system) factors may encourage or discourage Black men’s engagement in their health, which may be even more complicated in the case of prostate cancer as it touches on men’s masculine identity. The articles in this review primarily focused on intrapersonal [17, 18] and interpersonal factors [19] related to negative psychological responses [17] and uncertainties of screening [18] as well as the role of social support in decision-making [19]. Screening recommendations for Black men historically have not upheld a shared decision-making model between patients and physicians [3]. The goal of screening is to reduce incidence of advanced cancer diagnosis and earlier detection of disease to reduce premature mortality. In addition to the inter/intrapersonal interventions discussed in this review, community and societal level factors should be considered in addressing screening concerns for Black men.

An example of a community level intervention to potentially address screening concerns for Black men is the Prostate Cancer Precision Prevention Program (PCP3) [34]. The goal of the program is to leverage community partnerships and provide free PSA blood testing to at-risk non-Hispanic Black and Hispanic/Latino men in the greater Los Angeles catchment area (2017–2022) and the Atlanta Metropolitan area as well as rural Georgia (2023 to present). The program aims to decrease barriers to accessing prostate cancer screenings for Black and Hispanic/Latino men which may lead to earlier detection for prostate cancer. Screenings are provided to target populations at health education events and co-sponsored with community-based organizations (e.g., churches, grassroot advocacy groups, cancer survivor programs, community clinics). Memorandums of understanding with several community hospitals and free clinics created a referral network for under resourced participants who lacked access to a comprehensive cancer center. Additionally, policy initiatives like the PSA Screening for HIM Act or the Prostate-Specific Antigen Screening for High-risk Insured Men Act may reduce barriers to accessing care for at risk communities including non-Hispanic Black men [35]. Sadly, the U.S. Preventive Services Task Force screening recommendation for prostate cancer is between age 55 and 69, while other organizations recommend lower ages (40–45 years) of screening for men at higher risk of the disease [3]. This has resulted in provider confusion and grey areas to support and cover earlier screening. The act will require insurance companies to cover screenings for men at high risk (with a family history of prostate cancer) without any cost-sharing requirements. Clearly, addressing screening barriers for non-Hispanic Black men will require multilevel socioecological approaches, informed by best evidence, that will require inclusion of the target population in intervention ideations, implementation, and dissemination efforts[36].

### Limitations and Strengths

This systematic review should be interpreted in the context of a few limitations. The lack of rigorously conducted interventions available for this analysis emphasizes the need for future tailored clinical trials to determine best practices to increase prostate cancer screening participation among Black men in the U.S. Furthermore, two of the three studies included other racial/ethnic groups which provided comparison among different population groups. While this is informative, focusing on designing and evaluating interventions targeted to Black men that evaluate culturally informed interventions may increase our understanding of Black men’s attitudes and beliefs about prostate cancer screening participation. Despite these limitations, this systematic literature review contributes to the existing literature related to the understanding of prostate cancer screening clinical trial participation and highlights opportunities for future healthcare and community-based interventions, especially as it points to the urgent need for rigorously conducted clinical trials to improve increasing rates of prostate cancer in Black men.

## Conclusion

Prostate cancer is the second leading cause of death for men in the U.S. [1] and Black men have an increased mortality rate compared to their non-Hispanic white counterparts [12]. However, prostate cancer is preventable and treatable if diagnosed at an early stage. The use of clinical trials may provide a better understanding of obstacles that deter Black men from participating in prostate cancer screening. Understanding their concerns and barriers can provide context and guidance for future interventions that addresses their concerns about discrimination, cost of health services, time away from work, and societal norms about masculinity in relation to prostate cancer screening. Our analysis of the three articles suggest that a multifactorial approach is required to address disparities faced by Black men in the context of prostate cancer screening. It is essential to continue research on how Black men make decisions to participate in screening as well as identify methods to address mistrust in the healthcare system, strategies to strengthen communication with providers, and generate an inclusive patient-centered environment for Black men to discuss their health.

## Supplementary Information

Below is the link to the electronic supplementary material.

Supplementary file1 (DOCX 41 KB)

## Data Availability

This is a review of other studies using the PRISMA checklist for systematic reviews. No original data was collected for this paper.

## References

[1] American Cancer Society. Key Statistics for prostate cancer. https://www.cancer.org/cancer/types/prostate-cancer/about/key-statistics.html. Accessed Aug 2023

[2] Jemal A, Culp MB, Ma J, Islami F, Fedewa SA. Prostate cancer incidence 5 years after US Preventive Services Task Force recommendations against screening. JNCI: J Natl Cancer Inst. 2021;113(1):64–71. 32432713 10.1093/jnci/djaa068 PMC7781461

[3] Johnson JR et al. The complex interplay of modifiable risk factors affecting prostate cancer disparities in African American men. Nat Rev Urol. 2024. 10.1038/s41585-023-00849-5. 10.1038/s41585-023-00849-5 PMC11904840 38307952

[4] J. R. Novak, T. Peak, J. Gast, and M. Arnell, Associations between masculine norms and health-care utilization in highly religious, heterosexual men. Am J Men’s Health. 2019;13(3):1557988319856739. https://www.ncbi.nlm.nih.gov/pmc/articles/PMC6560804/pdf/10.1177_1557988319856739.pdf 10.1177/1557988319856739 PMC6560804 31184245

[5] American Cancer Society. Cancer Facts & Figures. 2023. https://www.cancer.org/research/cancer-facts-statistics/all-cancer-facts-figures/2023-cancer-facts-figures.html. Accessed August 2023

[6] Centers for Disease Control and Prevention. What are the benefits and harms of screening? https://www.cdc.gov/cancer/prostate/basic_info/benefits-harms.htm. Accessed July 2023

[7] National Institutes of Health. Crunching numbers: what cancer screening statistics really tell us. https://www.cancer.gov/about-cancer/screening/research/what-screening-statistics-mean. Accessed August 2023

[8] National Institutes of Health. Why should i participate in a clinical trial? https://www.nih.gov/health-information/nih-clinical-research-trials-you/why-should-i-participate-clinical-trial. Accessed July 2023

[9] Woods-Burnham L, Johnson JR, Hooker SE Jr, Bedell FW, Dorff TB, Kittles RA. The Role of diverse populations in US clinical trials. Med. 2021;2(1):21–4. 10.1016/j.medj.2020.12.009. 35590131 10.1016/j.medj.2020.12.009

[10] Woods-Burnham L. Not all champions are allies in health disparities research. Cell. 2020;183(3):580–2. 10.1016/j.cell.2020.09.045. 33125884 10.1016/j.cell.2020.09.045

[11] Zeigler-Johnson C, McDonald AC, Pinheiro P, Lynch S, Taioli E, Joshi S, Alpert N, Baudin J, Joachim C, Deloumeaux J, Oliver J, Bhakkan-Mambir B, Beaubrun-Renard M, Ortiz AG, Ragin C. Trends in prostate cancer incidence among black men in the Caribbean and the United States. Prostate. 2023;83(12):1207–16. 10.1002/pros.24580. 10.1002/pros.24580 PMC11256998 37244749

[12] Hinata N, Fujisawa M. Racial differences in prostate cancer characteristics and cancer-specific mortality: an overview. World J Men's Health. 2022;40(2):217–27. 10.5534/wjmh.210070. 10.5534/wjmh.210070 PMC8987139 35021294

[13] Jacobs EA, Rolle I, Ferrans CE, Whitaker EE, Warnecke RB. Understanding African Americans’ views of the trustworthiness of physicians. J Gen Intern Med. 2006;21(6):642–7. 10.1111/j.1525-1497.2006.00485.x. 16808750 10.1111/j.1525-1497.2006.00485.x PMC1924632

[14] Wells L, Gowda A. A legacy of mistrust: African Americans and the US healthcare system. Proc UCLA Health. 2020;24:1–3.

[15] Scharff DP, Mathews KJ, Jackson P, Hoffsuemmer J, Martin E, Edwards D. More than Tuskegee: understanding mistrust about research participation. J Health Care Poor Underserved. 2010;21(3):879–97. 10.1353/hpu.0.0323. 20693733 10.1353/hpu.0.0323 PMC4354806

[16] Powell W, Richmond J, Mohottige D, Yen I, Joslyn A, Corbie-Smith G. Medical mistrust, racism, and delays in preventive health screening among African-American men. Behav Med. 2019;45(2):102–17. 10.1080/08964289.2019.1585327. 31343960 10.1080/08964289.2019.1585327 PMC8620213

[17] Roussi P, et al. Effects of a randomized trial comparing standard and enhanced counseling for men at high risk of prostate cancer as a function of race and monitoring style. J Health Psychol. 2018;23(14):1800–9. 10.1177/1359105316671188. 28810355 10.1177/1359105316671188 PMC5561513

[18] Carlson DS, Grivas P, Wei W, Dhillon PK, Abraksia S. The effectiveness of shared compared to informed decision making for prostate cancer screening in a high-risk African American population: a randomized control trial. Cancer Investig. 2021;39(2):124–32. 10.1080/07357907.2020.1855441. 33410359 10.1080/07357907.2020.1855441

[19] Holt CL, et al. Can Women facilitate men’s prostate cancer screening informed decision-making? The M-PACT trial. J Health Commun. 2017;22(12):964–73. 10.1080/10810730.2017.1382616. 29173037 10.1080/10810730.2017.1382616

[20] Shungu N, Sterba KR. Barriers and facilitators to informed decision-making about prostate cancer screening among Black men. J Am Board Fam Med. 2021;34(5):925–36. 10.3122/jabfm.2021.05.210149. 34535518 10.3122/jabfm.2021.05.210149

[21] Page MJ, et al. The PRISMA 2020 statement: an updated guideline for reporting systematic reviews. BMJ. 2021;372:n71. 10.1136/bmj.n71. 33782057 10.1136/bmj.n71 PMC8005924

[22] Babineau J. Product review: covidence (systematic review software). J Can Health Libr Assoc/J de l’Assoc des bibliothèques de la santé du Canada. 2014;35(2):68–71. 10.5596/c14-016.

[23] Critical Appraisal Skills Programme. CASP randomised controlled trial standard checklist. https://casp-uk.net/images/checklist/documents/CASP-Randomised-Controlled-Trial-Checklist/CASP-RCT-Checklist-PDF.pdf. Accessed Jan 2023

[24] O. D. Center. Sample tool: stage of decision making (pre-autologous donation). https://decisionaid.ohri.ca/docs/develop/Tools/Stage_Decision_Making_PAD.pdf. Accessed 2023

[25] Lillard JW Jr, Moses KA, Mahal BA, George DJ. Racial disparities in Black men with prostate cancer: a literature review. Cancer. 2022;128(21):3787–95. 10.1002/cncr.34433. 36066378 10.1002/cncr.34433 PMC9826514

[26] Rogers CR, et al. Attitudes Toward genomic testing and prostate cancer research among Black men. Am J Prev Med. 2018;55(5 Suppl 1):S103-s111. 10.1016/j.amepre.2018.05.028. 30670195 10.1016/j.amepre.2018.05.028 PMC6352989

[27] Fong J, Venables M, D’Souza D, Maskerine C. Patient communication preferences for prostate cancer screening discussions: a scoping review. Ann Fam Med. 2023;21(5):448–55. 10.1370/afm.3011. 37748915 10.1370/afm.3011 PMC10519764

[28] Mitchell JA, Perry R. Disparities in patient-centered communication for Black and Latino men in the US cross-sectional results from the 2010 health and retirement study. PLoS One. 2020; 15(9): e0238356. 10.1371/journal.pone.0238356 10.1371/journal.pone.0238356 PMC7523955 32991624

[29] Ward P. Trust and communication in a doctor-patient relationship: a literature review. Arch Med. 2018;3(3):36.

[30] Bazargan M, Cobb S, Assari S. Discrimination and medical mistrust in a racially and ethnically diverse sample of California adults, (in eng). Ann Fam Med. 2021;19(1):4–15. 10.1370/afm.2632. 33431385 10.1370/afm.2632 PMC7800756

[31] McKenzie SK, Collings S, Jenkin G, River J. Masculinity, Social connectedness, and mental health: men’s diverse patterns of practice. Am J Mens Health. 2018;12(5):1247–61. 10.1177/1557988318772732. 29708008 10.1177/1557988318772732 PMC6142169

[32] DeAngelis T. Black men’s mental health matters. https://www.apa.org/monitor/2021/09/ce-black-mental-health. Accessed Nov 2023.

[33] Luque JS, Ross L, Gwede CK. Qualitative systematic review of barber-administered health education, promotion, screening and outreach programs in African-American communities. J Community Health. 2014;39(1):181–90. 10.1007/s10900-013-9744-3. 23913106 10.1007/s10900-013-9744-3 PMC3947222

[34] COH Cancer Center Community Outreach and Engagement. Community Inititiatives: Cancer Screening & Prevention Program. https://www.cityofhope.org/research/beckman-research-institute/population-sciences/health-equities/coe/community-initiatives. Accessed 2024

[35] American Cancer Society. The PSA screening for HIM Act (H.R. 1826/S. 2821). https://www.fightcancer.org/policy-resources/psa-screening-him-act-hr-1826s-2821. Accessed 2024

[36] McDougall JA, Hastert TA, Teteh DK, Rogers CR, Moss JL, Ochoa-Dominguez CY, Chebli P, Sutton AL, Qin B, Warner ET, Xiong S. Addressing social risks to accelerate health equity in cancer prevention and control. Cancer Epidemiol Biomarkers Prev. 2024;33(2):337–40. 10.1158/1055-9965.EPI-23-1212. 10.1158/1055-9965.EPI-23-1212 PMC12449779 38317629

