# Correction to: Evidence-based Prostate Cancer Screening Interventions for Black Men: A Systematic Review

**DOI:** 10.1007/s40615-024-02100-2

**Published:** 2024-07-26

**Authors:** Abigail Lopez, Jared T. Bailey, Dorothy Galloway, Leanne Woods-Burnham, Susanne B. Montgomery, Rick Kittles, Dede K. Teteh-Brooks

**Affiliations:** 1 Department of Health Sciences, Crean College of Health and Behavioral Sciences, Chapman University, One University Drive, Orange, CA USA; 2 Gangarosa Department of Environmental Health, Rollins School of Public Health, Emory University, Atlanta, GA USA; 3 River Oak Center for Children, Sacramento, CA USA; 4 Department of Surgery, Morehouse School of Medicine, Atlanta, GA USA; 5 School of Behavioral Health, Loma Linda University, Loma Linda, CA USA; 6 Department of Community Health and Preventive Medicine, Morehouse School of Medicine, Atlanta, GA USA

**Correction to: Journal of Racial and Ethnic Health Disparities**

10.1007/s40615-024-02085-y

Affiliation 4 was incorrectly assigned to coauthor Leanne Woods-Burnham in the article as originally published.

The original article has been corrected.

