# Supplementary material for: Evidence-based Prostate Cancer Screening Interventions for Black Men: A Systematic Review
Source: J Racial Ethn Health Disparities. 2024 Jul 10;12(4):2734–42. doi: 10.1007/s40615-024-02085-y (PMC11886399; doi:10.1007/s40615-024-02085-y)
Supplement: Supplementary file 1 — Supplementary file1 (DOCX 41 KB) [file 40615_2024_2085_MOESM1_ESM.docx]

**Appendix A: First search strategy**

Databases:

PubMed: 112

Ovid MEDLINE: 123

CINAHL Plus: 25

PsycInfo: 20

Total: 280

Duplicate records removed in EndNote 20 (Desktop): 119

**Total unique records: 161**

**Uploaded to Covidence on 6/29/2022 – 84 unique studies added (77 duplicates removed by Covidence based on previously imported references)**

**PubMed**

Search Strategy

("African Americans"[mh] OR "African ancestry"[tiab] OR "African Continental Ancestry Group"[tiab] OR "African descendant*"[tiab] OR "Blacks"[mh] OR Black*[tiab] OR “Black American”[tiab] OR “afro American”[tiab] OR “afro-american”[tiab])

AND

(prostat*[tiab] OR "Prostate"[mh] OR “Prostatic Neoplasms”[mh] OR “prostat* cancer*”[tiab] OR “cancer of the prostate”[tiab])

AND

("cancer screening*"[tiab] OR “screening*”[tiab] OR "Mass Screening"[mh] OR "cancer detection"[tiab] OR "cancer prevention"[tiab] OR “epidemiology”[mh] OR "Public Health Surveillance"[mh] OR "Early Diagnosis"[mh] OR "Preventive Health Services"[mh] OR “preventive health care”[tiab] OR "Digital Rectal Examination"[mh] OR "Prostate-Specific Antigen"[mh] OR PSA[tiab] OR "health education"[mh] OR "Research Subjects"[mh] OR "Clinical Trials as Topic"[mh])

AND

("clinical trial"[pt] OR "randomized controlled trial"[pt] OR "controlled clinical trial"[pt] OR "comparative study"[pt] OR "evaluation study"[pt] OR "multicenter study"[pt] OR "pragmatic clinical trial"[pt] OR "randomized"[tiab] OR ("randomly"[tiab] OR "trial"[tiab] OR "groups"[tiab])) NOT ("animals"[mh] NOT "humans"[mh])

AND

(2019/1/1:2022/6/28[pdat])

Search History

| **Search #** | **Query** | **Results** |
| --- | --- | --- |
| #1 | Search: **("African Americans"[mh] OR "African ancestry"[tiab] OR "African Continental Ancestry Group"[tiab] OR "African descendant*"[tiab] OR "Blacks"[mh] OR Black*[tiab] OR "Black American"[tiab] OR "afro American"[tiab] OR "afro-american"[tiab])** | 237,365 |
| #2 | Search: **(prostat*[tiab] OR "Prostate"[mh] OR "Prostatic Neoplasms"[mh] OR "prostat* cancer*"[tiab] OR "cancer of the prostate"[tiab])** | 255,702 |
| #3 | Search: **("cancer screening*"[tiab] OR "screening*"[tiab] OR "Mass Screening"[mh] OR "cancer detection"[tiab] OR "cancer prevention"[tiab] OR "epidemiology"[mh] OR "Public Health Surveillance"[mh] OR "Early Diagnosis"[mh] OR "Preventive Health Services"[mh] OR "preventive health care"[tiab] OR "Digital Rectal Examination"[mh] OR "Prostate-Specific Antigen"[mh] OR PSA[tiab] OR "health education"[mh] OR "Research Subjects"[mh] OR "Clinical Trials as Topic"[mh])** | 1,690,063 |
| #4 | Search: **#1 AND #2 AND #3** | 1,488 |
| #5 | Search: **("clinical trial"[pt] OR "randomized controlled trial"[pt] OR "controlled clinical trial"[pt] OR "comparative study"[pt] OR "evaluation study"[pt] OR "multicenter study"[pt] OR "pragmatic clinical trial"[pt] OR "randomized"[tiab] OR ("randomly"[tiab] OR "trial"[tiab] OR "groups"[tiab])) NOT ("animals"[mh] NOT "humans"[mh])** | 4,506,298 |
| #6 | Search: **#4 AND #5** | 685 |
| #7 | Search: **(2019/1/1:2022/6/28[pdat])** | 5,137,390 |
| #8 | Search: **#6 AND #7** | 112 |

Date: 6/28/2022

Number of results: 112

**Ovid MEDLINE and Epub Ahead of Print, In-Process, In-Data-Review & Other Non-Indexed Citations, Daily and Versions**

Search Strategy

randomized controlled trial.pt.

controlled clinical trial.pt.

pragmatic clinical trial.pt.

comparative study.pt.

evaluation study.pt.

multicenter study.pt.

randomized.ab.

randomly.ab.

trial.ab.

groups.ab.

1 or 2 or 3 or 4 or 5 or 6 or 7 or 8 or 9 or 10

exp animals/ not humans.sh.

11 not 12

AND

african americans/ OR ("african ancestry" OR "african continental ancestry group" OR "african descendant*" OR black* OR "black american" OR "afro american" OR "afro-american").ti,ab.

AND

prostate/ OR prostat*.ti,ab. OR exp prostatic neoplasms/ OR (prostat* ADJ4 cancer*).ti,ab.

AND

exp early diagnosis/ OR "cancer screening*".ti,ab. OR screening*.ti,ab. OR exp mass screening/ OR (cancer* ADJ4 detection).ti,ab. OR (cancer* ADJ4 prevention).ti,ab. OR public health surveillance/ OR watchful waiting/ OR digital rectal examination/ OR prostate-specific antigen/ OR PSA.ti,ab. OR "risk assessment".ti,ab. OR exp health education/ OR "personal risk*".ti,ab. OR exp risk factors/ OR exp preventive health services/ OR exp research subjects/ OR exp clinical trials as topic/ OR (trial ADJ4 participa*).ti,ab. OR (trial ADJ4 recruit*).ti,ab.

AND

limit # to yr="2019 -Current"

Search History

| **Search #** | **Query** | **Results** |
| --- | --- | --- |
| 1 | randomized controlled trial.pt. | 571786 |
| 2 | controlled clinical trial.pt. | 94920 |
| 3 | pragmatic clinical trial.pt. | 2112 |
| 4 | comparative study.pt. | 1911235 |
| 5 | evaluation study.pt. | 261699 |
| 6 | multicenter study.pt. | 322975 |
| 7 | randomized.ab. | 566563 |
| 8 | randomly.ab. | 385580 |
| 9 | trial.ab. | 606038 |
| 10 | groups.ab. | 2371823 |
| 11 | 1 or 2 or 3 or 4 or 5 or 6 or 7 or 8 or 9 or 10 | 5147144 |
| 12 | exp animals/ not humans.sh. | 5022183 |
| 13 | 11 not 12 | 4280274 |
| 14 | african americans/ or ("african ancestry" or "african continental ancestry group" or "african descendant*" or black* or "black american" or "afro american" or "afro-american").ti,ab. | 215810 |
| 15 | prostate/ or prostat*.ti,ab. or exp prostatic neoplasms/ or (prostat* adj4 cancer*).ti,ab. | 254672 |
| 16 | exp early diagnosis/ or "cancer screening*".ti,ab. or screening*.ti,ab. or exp mass screening/ or (cancer* adj4 detection).ti,ab. or (cancer* adj4 prevention).ti,ab. or public health surveillance/ or watchful waiting/ or digital rectal examination/ or prostate-specific antigen/ or PSA.ti,ab. or "risk assessment".ti,ab. or exp health education/ or "personal risk*".ti,ab. or exp risk factors/ or exp preventive health services/ or exp research subjects/ or exp clinical trials as topic/ or (trial adj4 participa*).ti,ab. or (trial adj4 recruit*).ti,ab. | 2568024 |
| 17 | 14 and 15 and 16 | 1691 |
| 18 | 13 and 17 | 739 |
| 19 | limit 18 to yr="2019 -Current" | 123 |

Date:6/28/2022

Number of results: 123

**CINAHL Plus with Full Text (EBSCO)**

Search Strategy

MH “african americans”

OR

TI ("african ancestry" OR "african continental ancestry group" OR "african descendant*" OR black* OR "black american" OR "afro american" OR "afro-american")

OR

AB ("african ancestry" OR "african continental ancestry group" OR "african descendant*" OR black* OR "black american" OR "afro american" OR "afro-american")

AND

MH (“prostate” OR “prostatic neoplasms+”)

OR

TI (“prostat*” N3 “cancer*”)

OR

AB (“prostat*” N3 “cancer*”)

AND

MH (“cancer screening” OR “early diagnosis+” OR “epidemiology+” OR “preventive health care” OR “digital rectal examination” OR “Prostate-Specific Antigen” OR “health education” OR “research subjects” OR “research subject recruitment” OR “research subject retention”)

OR

TI (“diagnos*” OR “screening*” OR “mass screening*” OR (“cancer*” N3 “detection”) OR (“cancer*” N3 “prevention”) OR “preventive health services” OR “PSA” OR (“trial” N3 “participa*”) OR (“trial” N3 “recruit*”))

OR

AB (“diagnos*” OR “screening*” OR “mass screening*” OR (“cancer*” N3 “detection”) OR (“cancer*” N3 “prevention”) OR “preventive health services” OR “PSA” OR (“trial” N3 “participa*”) OR (“trial” N3 “recruit*”))

AND

(MH "Clinical Trials+") OR PT Clinical trial OR PT Randomized controlled trial OR TX (clinic* n1 trial*) OR TX ((singl* n1 blind*) or (singl* n1 mask*)) OR TX ( (doubl* n1 blind*) or (doubl* n1 mask*)) or TX ((tripl* n1 blind*) or (tripl* n1 mask*)) OR TX (randomi* control* trial*) OR MH "Random Assignment" OR TX random* allocat* OR MH "Quantitative Studies" OR TX allocat* random*

AND

PY 2019-

Search History

| **Search #** | **Query** | **Results** |
| --- | --- | --- |
| S1 | (MH "Clinical Trials+") | 339,868 |
| S2 | PT Clinical trial | 112,824 |
| S3 | PT Randomized controlled trial | 143,678 |
| S4 | TX clinic* n1 trial* | 396,710 |
| S5 | TX ((singl* n1 blind*) or (singl* n1 mask*)) or TX ( (doubl* n1 blind*) or (doubl* n1 mask*)) or TX ((tripl* n1 blind*) or (tripl* n1 mask*)) | 1,276,743 |
| S6 | TX randomi* control* trial* | 290,809 |
| S7 | (MH "Random Assignment") | 74,377 |
| S8 | TX random* allocat* | 16,506 |
| S9 | (MH "Quantitative Studies") | 32,267 |
| S10 | TX allocat* random* | 1,254 |
| S11 | S1 or S2 or S3 or S4 or S5 or S6 or S7 or S8 or S9 or S10 | 1,745,488 |
| S12 | MH “african americans” OR TI ("african ancestry" OR "african continental ancestry group" OR "african descendant*" OR black* OR "black american" OR "afro american" OR "afro-american") OR AB ("african ancestry" OR "african continental ancestry group" OR "african descendant*" OR black* OR "black american" OR "afro american" OR "afro-american") | 50,037 |
| S13 | MH (“prostate” OR “prostatic neoplasms+”) OR TI (“prostat*” N3 “cancer*”) OR AB (“prostat*” N3 “cancer*”) | 44,837 |
| S14 | MH (“cancer screening” OR “early diagnosis+” OR “epidemiology+” OR “preventive health care” OR “digital rectal examination” OR “Prostate-Specific Antigen” OR “health education” OR “research subjects” OR “research subject recruitment” OR “research subject retention”) OR TI (“diagnos*” OR “screening*” OR “mass screening*” OR (“cancer*” N3 “detection”) OR (“cancer*” N3 “prevention”) OR “preventive health services” OR “PSA” OR (“trial” N3 “participa*”) OR (“trial” N3 “recruit*”)) OR AB (“diagnos*” OR “screening*” OR “mass screening*” OR (“cancer*” N3 “detection”) OR (“cancer*” N3 “prevention”) OR “preventive health services” OR “PSA” OR (“trial” N3 “participa*”) OR (“trial” N3 “recruit*”)) | 1,452,413 |
| S15 | S12 and S13 and S14 | 443 |
| S16 | S15 and S11 | 108 |
| S17 | PY 2019- | 1,572,111 |
| S18 | S16 and S17 | 25 |

Date: 6/28/2022

Number of results: 25

**PsycInfo (EBSCO)**

Search Strategy

DE Blacks

OR

TI (“african american” OR "african ancestry" OR "african continental ancestry group" OR "african descendant*" OR black* OR "black american" OR "afro american" OR "afro-american")

OR

AB (“african american” OR "african ancestry" OR "african continental ancestry group" OR "african descendant*" OR black* OR "black american" OR "afro american" OR "afro-american")

AND

DE prostate

OR

TI ((“prostat*” N3 “cancer*”) or “prostatic neoplasms”)

OR

AB ((“prostat*” N3 “cancer*”) or “prostatic neoplasms”)

AND

DE (“cancer screening” OR “medical diagnosis” OR “epidemiology+” OR “preventive health services+” OR “health education+” OR “experimental subjects” OR “experimental recruitment”)

OR

TI (“diagnos*” OR “screening*” OR “mass screening*” OR (“cancer*” N3 “detection”) OR (“cancer*” N3 “prevention”) OR “PSA” OR “digital rectal examination” OR “Prostate-Specific Antigen” OR (“trial” N3 “participa*”) OR (“trial” N3 “recruit*”))

OR

AB (“diagnos*” OR “screening*” OR “mass screening*” OR (“cancer*” N3 “detection”) OR (“cancer*” N3 “prevention”) OR “PSA” OR “digital rectal examination” OR “Prostate-Specific Antigen” OR (“trial” N3 “participa*”) OR (“trial” N3 “recruit*”))

AND

DE Clinical Trials+ or MR clinical trial or TX (clinic* n1 trial*) or TX ((singl* n1 blind*) or (singl* n1 mask*)) or TX ((doubl* n1 blind*) or (doubl* n1 mask*)) or TX ((tripl* n1 blind*) or (tripl* n1 mask*)) or TX (randomi* control* trial*) or DE Random Sampling or TX (random* allocat*) or TX (random* allocat*) or MR quantitative study or TX (allocat* random*)

AND

PY 2019-

Search History

| **Search #** | **Query** | **Results** |
| --- | --- | --- |
| S1 | DE Blacks OR TI (“african american” OR "african ancestry" OR "african continental ancestry group" OR "african descendant*" OR black* OR "black american" OR "afro american" OR "afro-american") OR AB (“african american” OR "african ancestry" OR "african continental ancestry group" OR "african descendant*" OR black* OR "black american" OR "afro american" OR "afro-american") | 116,153 |
| S2 | DE prostate OR TI ((“prostat*” N3 “cancer*”) or “prostatic neoplasms”) OR AB ((“prostat*” N3 “cancer*”) or “prostatic neoplasms”) | 3,931 |
| S3 | DE (“cancer screening” OR “medical diagnosis” OR “epidemiology+” OR “preventive health services+” OR “health education+” OR “experimental subjects” OR “experimental recruitment”) OR TI (“diagnos*” OR “screening*” OR “mass screening*” OR (“cancer*” N3 “detection”) OR (“cancer*” N3 “prevention”) OR “PSA” OR “digital rectal examination” OR “Prostate-Specific Antigen” OR (“trial” N3 “participa*”) OR (“trial” N3 “recruit*”)) OR AB (“diagnos*” OR “screening*” OR “mass screening*” OR (“cancer*” N3 “detection”) OR (“cancer*” N3 “prevention”) OR “PSA” OR “digital rectal examination” OR “Prostate-Specific Antigen” OR (“trial” N3 “participa*”) OR (“trial” N3 “recruit*”)) | 411,413 |
| S4 | S1 AND S2 AND S3 | 336 |
| S5 | DE Clinical Trials+ or MR clinical trial or TX (clinic* n1 trial*) or TX ((singl* n1 blind*) or (singl* n1 mask*)) or TX ((doubl* n1 blind*) or (doubl* n1 mask*)) or TX ((tripl* n1 blind*) or (tripl* n1 mask*)) or TX (randomi* control* trial*) or DE Random Sampling or TX (random* allocat*) or TX (random* allocat*) or MR quantitative study or TX (allocat* random*) | 1,881,995 |
| S6 | S4 and S5 | 244 |
| S7 | PY 2019- | 586,563 |
| S8 | S6 and S7 | 26 |
| S9 | PT Journal | 4,117,546 |
| S10 | S8 and S9 | 20 |

Date: 6/28/2022

Number of results: 20

**Appendix B: Second search strategy**

Updated search on January 10, 2023

Search expanded to include date range of 1/1/2017-1/10/2023

Databases:

PubMed: 174

Ovid MEDLINE: 206

CINAHL Plus: 62

PsycInfo: 48

Total: 490

Duplicate records removed in EndNote 20 (Desktop) (including duplicates with original 161  records): 357

**Total records: 133**

**Uploaded to Covidence on 1/10/2023 – 127 unique studies added (6 duplicates removed by  Covidence based on previously imported references)**

**PubMed**

Search Strategy

("Black or African American"[mh] OR "African ancestry"[tiab] OR "African Continental  Ancestry Group"[tiab] OR "African descendant*"[tiab] OR Black*[tiab] OR “Black  American”[tiab] OR “afro American”[tiab] OR “afro-american”[tiab])

AND

(prostat*[tiab] OR "Prostate"[mh] OR “Prostatic Neoplasms”[mh] OR “prostat* cancer*”[tiab]  OR “cancer of the prostate”[tiab])

AND

("cancer screening*"[tiab] OR “screening*”[tiab] OR "Mass Screening"[mh] OR "cancer  detection"[tiab] OR "cancer prevention"[tiab] OR “epidemiology”[mh] OR "Public Health  Surveillance"[mh] OR "Early Diagnosis"[mh] OR "Preventive Health Services"[mh] OR  “preventive health care”[tiab] OR "Digital Rectal Examination"[mh] OR "Prostate-Specific  Antigen"[mh] OR PSA[tiab] OR "health education"[mh] OR "Research Subjects"[mh] OR  "Clinical Trials as Topic"[mh])

AND

("clinical trial"[pt] OR "randomized controlled trial"[pt] OR "controlled clinical trial"[pt] OR  "comparative study"[pt] OR "evaluation study"[pt] OR "multicenter study"[pt] OR "pragmatic  clinical trial"[pt] OR "randomized"[tiab] OR ("randomly"[tiab] OR "trial"[tiab] OR  "groups"[tiab])) NOT ("animals"[mh] NOT "humans"[mh])

AND

(2017/1/1:2023/1/10[pdat])

Search History

| Search # | Query | Results |
| --- | --- | --- |
| 1 | **("Black or African American"[mh] OR "African ancestry"[tiab]  OR "African Continental Ancestry Group"[tiab] OR "African  descendant*"[tiab] OR Black*[tiab] OR "Black American"[tiab]  OR "afro American"[tiab] OR "afro-american"[tiab])** | 226,427 |
| 2 | **(prostat*[tiab] OR "Prostate"[mh] OR "Prostatic**  **Neoplasms"[mh] OR "prostat* cancer*"[tiab] OR "cancer of the  prostate"[tiab])** | 261,956 |
| 3 | **("cancer screening*"[tiab] OR "screening*"[tiab] OR "Mass  Screening"[mh] OR "cancer detection"[tiab] OR "cancer  prevention"[tiab] OR "epidemiology"[mh] OR "Public Health  Surveillance"[mh] OR "Early Diagnosis"[mh] OR "Preventive  Health Services"[mh] OR "preventive health care"[tiab] OR  "Digital Rectal Examination"[mh] OR "Prostate-Specific  Antigen"[mh] OR PSA[tiab] OR "health education"[mh] OR  "Research Subjects"[mh] OR "Clinical Trials as Topic"[mh])** | 1,741,977 |
| 4 | **#1 AND #2 AND #3** | 1,379 |
| 5 | **("clinical trial"[pt] OR "randomized controlled trial"[pt] OR  "controlled clinical trial"[pt] OR "comparative study"[pt] OR  "evaluation study"[pt] OR "multicenter study"[pt] OR  "pragmatic clinical trial"[pt] OR "randomized"[tiab] OR  ("randomly"[tiab] OR "trial"[tiab] OR "groups"[tiab])) NOT  ("animals"[mh] NOT "humans"[mh])** | 4,624,619 |
| 6 | **#4 AND #5** | 631 |
| 7 | **(2017/1/1:2023/1/10[pdat])** | 8,268,385 |
| 8 | **#6 AND #7** | 174 |

Date: 1/10/2023

Number of results: 174

**Ovid MEDLINE and Epub Ahead of Print, In-Process, In-Data-Review & Other Non Indexed Citations, Daily and Versions**

Search Strategy

randomized controlled trial.pt.

controlled clinical trial.pt.

pragmatic clinical trial.pt.

comparative study.pt.

evaluation study.pt.

multicenter study.pt.

randomized.ab.

randomly.ab.

trial.ab.

groups.ab.

1 or 2 or 3 or 4 or 5 or 6 or 7 or 8 or 9 or 10

exp animals/ not humans.sh.

11 not 12

AND

african americans/ OR ("african ancestry" OR "african continental ancestry group" OR "african  descendant*" OR black* OR "black american" OR "afro american" OR "afro-american").ti,ab.

AND

prostate/ OR prostat*.ti,ab. OR exp prostatic neoplasms/ OR (prostat* ADJ4 cancer*).ti,ab. AND

exp early diagnosis/ OR "cancer screening*".ti,ab. OR screening*.ti,ab. OR exp mass screening/  OR (cancer* ADJ4 detection).ti,ab. OR (cancer* ADJ4 prevention).ti,ab. OR public health  surveillance/ OR watchful waiting/ OR digital rectal examination/ OR prostate-specific antigen/  OR PSA.ti,ab. OR "risk assessment".ti,ab. OR exp health education/ OR "personal risk*".ti,ab.  OR exp risk factors/ OR exp preventive health services/ OR exp research subjects/ OR exp  clinical trials as topic/ OR (trial ADJ4 participa*).ti,ab. OR (trial ADJ4 recruit*).ti,ab.

AND

limit # to yr="2017 -Current"

Search History

| **Search #** | **Query** | **Results** |
| --- | --- | --- |
| 1 | randomized controlled trial.pt. | 584037 |
| 2 | controlled clinical trial.pt. | 95153 |
| 3 | pragmatic clinical trial.pt. | 2173 |
| 4 | comparative study.pt. | 1911883 |
| 5 | evaluation study.pt. | 261750 |
| 6 | multicenter study.pt. | 329165 |
| 7 | randomized.ab. | 588506 |
| 8 | randomly.ab. | 399386 |
| 9 | trial.ab. | 630666 |
| 10 | groups.ab. | 2459478 |
| 11 | 1 or 2 or 3 or 4 or 5 or 6 or 7 or 8 or 9 or 10 | 5270141 |
| 12 | exp animals/ not humans.sh. | 5080261 |
| 13 | 11 not 12 | 4394478 |
| 14 | african americans/ or ("african ancestry" or "african continental  ancestry group" or "african descendant*" or black* or "black  american" or "afro american" or "afro-american").ti,ab. | 224621 |
| 15 | prostate/ or prostat*.ti,ab. or exp prostatic neoplasms/ or  (prostat* adj4 cancer*).ti,ab. | 260711 |
| 16 | exp early diagnosis/ or "cancer screening*".ti,ab. or  screening*.ti,ab. or exp mass screening/ or (cancer* adj4  detection).ti,ab. or (cancer* adj4 prevention).ti,ab. or public  health surveillance/ or watchful waiting/ or digital rectal  examination/ or prostate-specific antigen/ or PSA.ti,ab. or "risk  assessment".ti,ab. or exp health education/ or "personal  risk*".ti,ab. or exp risk factors/ or exp preventive health  services/ or exp research subjects/ or exp clinical trials as topic/  or (trial adj4 participa*).ti,ab. or (trial adj4 recruit*).ti,ab. | 2627683 |
| 17 | 14 and 15 and 16 | 1731 |
| 18 | 13 and 17 | 757 |
| 19 | limit 18 to yr="2017 -Current" | 206 |

Date: 1/10/2023

Number of results: 206

**CINAHL Plus with Full Text (EBSCO)**

Search Strategy

MH “african americans”

OR

TI ("african ancestry" OR "african continental ancestry group" OR "african descendant*" OR  black* OR "black american" OR "afro american" OR "afro-american")

OR

AB ("african ancestry" OR "african continental ancestry group" OR "african descendant*" OR  black* OR "black american" OR "afro american" OR "afro-american")

AND

MH (“prostate” OR “prostatic neoplasms+”)

OR

TI (“prostat*” N3 “cancer*”)

OR

AB (“prostat*” N3 “cancer*”)

AND

MH (“cancer screening” OR “early diagnosis+” OR “epidemiology+” OR “preventive health  care” OR “digital rectal examination” OR “Prostate-Specific Antigen” OR “health education”  OR “research subjects” OR “research subject recruitment” OR “research subject retention”) OR

TI (“diagnos*” OR “screening*” OR “mass screening*” OR (“cancer*” N3 “detection”) OR  (“cancer*” N3 “prevention”) OR “preventive health services” OR “PSA” OR (“trial” N3  “participa*”) OR (“trial” N3 “recruit*”))

OR

AB (“diagnos*” OR “screening*” OR “mass screening*” OR (“cancer*” N3 “detection”) OR  (“cancer*” N3 “prevention”) OR “preventive health services” OR “PSA” OR (“trial” N3  “participa*”) OR (“trial” N3 “recruit*”))

AND

(MH "Clinical Trials+") OR PT Clinical trial OR PT Randomized controlled trial OR TX  (clinic* n1 trial*) OR TX ((singl* n1 blind*) or (singl* n1 mask*)) OR TX ( (doubl* n1 blind*)  or (doubl* n1 mask*)) or TX ((tripl* n1 blind*) or (tripl* n1 mask*)) OR TX (randomi* control*  trial*) OR MH "Random Assignment" OR TX random* allocat* OR MH "Quantitative Studies"  OR TX allocat* random*

AND

PY 2017-

Search History

| **Search #** | **Query** | **Results** |
| --- | --- | --- |
| S1 | (MH "Clinical Trials+") | 348,294 |
| S2 | PT Clinical trial | 114,444 |
| S3 | PT Randomized controlled trial | 149,497 |
| S4 | TX clinic* n1 trial* | 408,733 |
| S5 | TX ((singl* n1 blind*) or (singl* n1 mask*)) or TX ( (doubl*  n1 blind*) or (doubl* n1 mask*)) or TX ((tripl* n1 blind*) or  (tripl* n1 mask*)) | 1,307,220 |
| S6 | TX randomi* control* trial* | 303,453 |
| S7 | (MH "Random Assignment") | 77,385 |
| S8 | TX random* allocat* | 17,201 |
| S9 | (MH "Quantitative Studies") | 34,001 |
| S10 | TX allocat* random* | 1,285 |
| S11 | S1 or S2 or S3 or S4 or S5 or S6 or S7 or S8 or S9 or S10 | 1,793,562 |
| S12 | MH “african americans” OR TI ("african ancestry" OR "african  continental ancestry group" OR "african descendant*" OR black* OR "black american" OR "afro american" OR "afro american") OR AB ("african ancestry" OR "african continental  ancestry group" OR "african descendant*" OR black* OR  "black american" OR "afro american" OR "afro-american") | 68,795 |
| S13 | MH (“prostate” OR “prostatic neoplasms+”) OR TI (“prostat*”  N3 “cancer*”) OR AB (“prostat*” N3 “cancer*”) | 46,484 |
| S14 | MH (“cancer screening” OR “early diagnosis+” OR  “epidemiology+” OR “preventive health care” OR “digital  rectal examination” OR “Prostate-Specific Antigen” OR  “health education” OR “research subjects” OR “research  subject recruitment” OR “research subject retention”) OR TI  (“diagnos*” OR “screening*” OR “mass screening*” OR  (“cancer*” N3 “detection”) OR (“cancer*” N3 “prevention”)  OR “preventive health services” OR “PSA” OR (“trial” N3  “participa*”) OR (“trial” N3 “recruit*”)) OR AB (“diagnos*”  OR “screening*” OR “mass screening*” OR (“cancer*” N3  “detection”) OR (“cancer*” N3 “prevention”) OR “preventive  health services” OR “PSA” OR (“trial” N3 “participa*”) OR  (“trial” N3 “recruit*”)) | 1,500,106 |
| S15 | S12 and S13 and S14 | 767 |
| S16 | S15 and S11 | 222 |
| S17 | PY 2017- | 2,690,452 |
| S18 | S16 and S17 | 62 |

Date: 1/10/2023

Number of results: 62

**PsycInfo (EBSCO)**

Search Strategy

DE Blacks

OR

TI (“african american” OR "african ancestry" OR "african continental ancestry group" OR  "african descendant*" OR black* OR "black american" OR "afro american" OR "afro-american") OR

AB (“african american” OR "african ancestry" OR "african continental ancestry group" OR "african descendant*" OR black* OR "black american" OR "afro american" OR "afro-american")

AND

DE prostate

OR

TI ((“prostat*” N3 “cancer*”) or “prostatic neoplasms”)

OR

AB ((“prostat*” N3 “cancer*”) or “prostatic neoplasms”)

AND

DE (“cancer screening” OR “medical diagnosis” OR “epidemiology+” OR “preventive health  services+” OR “health education+” OR “experimental subjects” OR “experimental recruitment”) OR

TI (“diagnos*” OR “screening*” OR “mass screening*” OR (“cancer*” N3 “detection”) OR  (“cancer*” N3 “prevention”) OR “PSA” OR “digital rectal examination” OR “Prostate-Specific  Antigen” OR (“trial” N3 “participa*”) OR (“trial” N3 “recruit*”))

OR

AB (“diagnos*” OR “screening*” OR “mass screening*” OR (“cancer*” N3 “detection”) OR  (“cancer*” N3 “prevention”) OR “PSA” OR “digital rectal examination” OR “Prostate-Specific  Antigen” OR (“trial” N3 “participa*”) OR (“trial” N3 “recruit*”))

AND

DE Clinical Trials+ or MR clinical trial or TX (clinic* n1 trial*) or TX ((singl* n1 blind*) or  (singl* n1 mask*)) or TX ((doubl* n1 blind*) or (doubl* n1 mask*)) or TX ((tripl* n1 blind*) or  (tripl* n1 mask*)) or TX (randomi* control* trial*) or DE Random Sampling or TX (random*  allocat*) or TX (random* allocat*) or MR quantitative study or TX (allocat* random*)

AND

PY 2017-

Search History

| **Search #** | **Query** | **Results** |
| --- | --- | --- |
| S1 | DE Blacks OR TI (“african american” OR "african ancestry" OR "african continental ancestry group" OR "african descendant*" OR black* OR "black american" OR "afro american" OR "afro-american") OR AB (“african american” OR "african ancestry" OR "african continental ancestry group" OR "african descendant*" OR black* OR "black american" OR "afro american" OR "afro-american") | 119,246 |
| S2 | DE prostate OR TI ((“prostat*” N3 “cancer*”) or “prostatic neoplasms”) OR AB ((“prostat*” N3 “cancer*”) or “prostatic neoplasms”) | 4,007 |
| S3 | DE (“cancer screening” OR “medical diagnosis” OR “epidemiology+” OR “preventive health services+” OR “health education+” OR “experimental subjects” OR “experimental recruitment”) OR TI (“diagnos*” OR “screening*” OR “mass screening*” OR (“cancer*” N3 “detection”) OR (“cancer*” N3 “prevention”) OR “PSA” OR “digital rectal examination” OR “Prostate-Specific Antigen” OR (“trial” N3 “participa*”) OR (“trial” N3 “recruit*”)) OR AB (“diagnos*” OR “screening*” OR “mass screening*” OR (“cancer*” N3 “detection”) OR (“cancer*” N3 “prevention”) OR “PSA” OR “digital rectal examination” OR “Prostate Specific Antigen” OR (“trial” N3 “participa*”) OR (“trial” N3 “recruit*”)) | 420,624 |
| S4 | S1 AND S2 AND S3 | 341 |
| S5 | DE Clinical Trials+ or MR clinical trial or TX (clinic* n1 trial*) or TX ((singl* n1 blind*) or (singl* n1 mask*)) or TX ((doubl* n1 blind*) or (doubl* n1 mask*)) or TX ((tripl* n1 blind*) or (tripl* n1 mask*)) or TX (randomi* control* trial*) or DE Random Sampling or TX (random* allocat*) or TX (random* allocat*) or MR quantitative study or TX (allocat* random*) | 1,942,700 |
| S6 | S4 and S5 | 250 |
| S7 | PY 2017- | 1,046,620 |
| S8 | S6 and S7 | 62 |
| S9 | PT Journal | 4,195,433 |
| S10 | S8 and S9 | 48 |

Date: 1/10/2023

Number of results: 48
